# Specific Interaction with Cardiolipin Triggers Functional Activation of Dynamin-Related Protein 1

**DOI:** 10.1371/journal.pone.0102738

**Published:** 2014-07-18

**Authors:** Itsasne Bustillo-Zabalbeitia, Sylvie Montessuit, Etienne Raemy, Gorka Basañez, Oihana Terrones, Jean-Claude Martinou

**Affiliations:** 1 Biophysics Unit (CSIC-UPV/EHU) and Department of Biochemistry and Molecular Biology, University of the Basque Country (UPV/EHU), Bilbao, Spain; 2 Department of Cell Biology, University of Geneva, Geneva, Switzerland; University of Iowa, United States of America

## Abstract

Dynamin-Related Protein 1 (Drp1), a large GTPase of the dynamin superfamily, is required for mitochondrial fission in healthy and apoptotic cells. Drp1 activation is a complex process that involves translocation from the cytosol to the mitochondrial outer membrane (MOM) and assembly into rings/spirals at the MOM, leading to membrane constriction/division. Similar to dynamins, Drp1 contains GTPase (G), bundle signaling element (BSE) and stalk domains. However, instead of the lipid–interacting Pleckstrin Homology (PH) domain present in the dynamins, Drp1 contains the so-called B insert or variable domain that has been suggested to play an important role in Drp1 regulation. Different proteins have been implicated in Drp1 recruitment to the MOM, although how MOM-localized Drp1 acquires its fully functional status remains poorly understood. We found that Drp1 can interact with pure lipid bilayers enriched in the mitochondrion-specific phospholipid cardiolipin (CL). Building on our previous study, we now explore the specificity and functional consequences of this interaction. We show that a four lysine module located within the B insert of Drp1 interacts preferentially with CL over other anionic lipids. This interaction dramatically enhances Drp1 oligomerization and assembly-stimulated GTP hydrolysis. Our results add significantly to a growing body of evidence indicating that CL is an important regulator of many essential mitochondrial functions.

## Introduction

Mitochondria are complex organelles where a variety of crucial processes required for correct regulation of cell physiology take place. It has long been known that under healthy conditions, mitochondria supply the cell with ATP produced by oxidative phosphorylation and participate in a variety of catabolic and anabolic pathways [1]. During the last two decades it also became recognized that mitochondria play an essential role in apoptosis. Diverse apoptotic stimuli cause release of apoptogenic factors from mitochondria that ultimately lead to caspase activation [2], [3].

Mitochondria are highly dynamic organelles that undergo fusion and fission events to a differing degree depending on the physiological status of the cell and on environmental cues [4], [5]. Abnormalities in mitochondrial fusion have been causatively linked to human neuropathies such as Charcot-Marie-Tooth disease type 2A [6] and Dominant Optic Atrophy [7], [8], while defects in mitochondrial fission have been implicated in Parkinson's disease [9], [10] and Alzheimer's disease [11]. Mitochondrial morphological dynamics are mainly regulated by dynamin-related GTPases, a group of proteins with the intrinsic capacity to reorganize membrane structure in a oligomerization- and GTP hydrolysis-dependent manner leading to structural reorganization of the mitochondrial membranes [5], [12]. In mammals the dynamin-related GTPases implicated in mitochondrial fusion are the inner membrane protein Optic atrophy 1 (Opa1) and the outer membrane proteins mitofusins 1 and 2 (Mfn1 and Mfn2) [13]. On the other hand, mitochondrial fission relies on Drp1, which is also involved in peroxisome division [14]–[17]. Ablation of Drp1 function in mice has revealed the importance of this protein for correct development of the embryonic brain and other tissues [18], [19]. Importantly, dynamin-related GTPases also play an essential role in the normal progression of apoptosis [20]. In this regard, we have shown that Drp1 stimulates Bax oligomerization and cytochrome c release by promoting tethering and hemifusion of mitochondrial membranes [21]. In addition, in healthy cells Opa1 has been shown to assemble into high-order oligomers to maintain the architecture of the mitochondrial cristae, while during apoptosis, Opa1 oligomers disassemble to allow effective release of cytochrome c [22], [23].

Drp1 shows a four-domain architecture composed of the GTPase domain (G), the bundle signaling element (BSE) implicated in the transmission of conformational changes from the G domain to the stalk, a so-called B insert implicated in regulation of Drp1 function and the stalk implicated in Drp1 multimerization [24]. Inactive Drp1 is predominantly cytosolic, although a subpool of the protein concentrates in specific patches on mitochondria at sites of future fission [15], presumably where endoplasmic reticulum tubules contact mitochondria [25], [26]. Different aspects of Drp1 function, including its localization, stability, and GTPase activity have been reported to be regulated by posttranslational modifications such as phosphorylation [27], SUMOylation [28], [29], ubiquitination [30], O-linked-N-acetyl-glucosamine glycosylation [31], and S-nitrosylation [11], [32]. Interestingly, most of these post-translational modifications occur at the B insert.

Based on studies performed with Dnm1, the yeast ortholog of Drp1, it was originally proposed that when Drp1 translocates to mitochondria, it docks onto the Fis1 protein, through an interaction with the adaptor protein Mdv1 or its paralogue Caf4 [12]. Although Fis1 is highly conserved throughout the eukaryotic kingdom including humans, mammalian homologues of Mdv1 and Caf4 have yet to be found. Moreover, recent evidence indicates that Fis1 regulates mitochondrial morphology in mammals by recruiting not Drp1, but a GTPase regulator domain-containing protein termed TBC1D15 [33]. In addition, in mammalian cells other mitochondrial proteins have been proposed to be essential for mitochondrial recruitment and functional regulation of Drp1. Two prominent examples are the mitochondrial fission factor (Mff) and the mitochondrial dynamics proteins of 51 (MiD51 or MIEF1) and 49 kDa (MiD49 or MIEF2), which have been implicated both in Drp1 mitochondrial recruitment and in stimulation of mitochondrial fission [34]–[36]. Importantly, neither Mff nor MiD49 can effectively increase the GTP hydrolysis activity of Drp1 as classical GTPase effectors do [34], therefore whether this interaction is in itself sufficient to trigger functional activation of Drp1 remains unproven.

In addition to proteins, specific lipids can also act as regulatory factors of classical dynamin and dynamin-related proteins. Classical dynamins contain a Pleckstrin Homology (PH) domain that interacts with polyanionic phosphatidylinositol bisphosphate (PIP_2_) to regulate protein assembly and function [37], [38]. On the other hand, dynamin-like proteins such as yeast Mgm1 and human Opa1 do not contain a recognizable PH domain, even though they have been shown to interact with the mitochondrion-specific polyanionic phospholipid cardiolipin (CL). Moreover the interaction of CL with Mgm1 and Opa1 stimulates GTPase activity [39]–[41]. CL thus appears to play a key role in mitochondrial function [42], [43]. CL is primarily localized at the inner mitochondrial membrane, although significant amounts are also found at the MOM, particularly at the so-called mitochondrial membrane contact sites [44]. Interestingly, during apoptosis Drp1 translocates from cytosol to MOM and co-localizes with Bax, which in turn is known to target to mitochondrial foci enriched in CL [45], [46]. Moreover, we have found that Drp1 interacts directly with liposomes containing CL [21]. Prompted by these observations, we have attempted to gain further insight into the molecular specificity and functional consequences of the Drp1:CL interaction. In this report, we provide evidence that Drp1 interacts specifically with CL through its B insert, and also show that this interaction promotes Drp1 oligomerization and stimulation of its GTPase activity.

## Materials and Methods

### Materials

Egg phosphatidylcholine (PC), egg phosphatidylethanolamine (PE), liver phosphatidylinositol (PI), heart cardiolipin (CL), brain phosphatydilserine (PS), and egg phosphatidyldglycerol (PG) were purchased from Avanti Polar Lipids, Inc. (Alabaster,AL). Anti Drp1 Abs. were from BD-Biosciences and Thermo Scientific. Biacore sensor chip L1 was purchased from GE Healthcase.

### Expression and Purification of Recombinant Proteins

Untagged Drp1 isoforms 1, 2 and 3 and the mutants in the B insert (K557-560A, K569-571A and K557-560-569-571A) were produced in *Escherichia coli* Rosetta cells. Cells were grown at 37°C in LB media containing 100 µg/ml ampicillin to reach an OD600 of 0.9 followed by induction with 0.5 mM isopropyl β-D-thiogalactopyranoside (IPTG) for 12 h at 18°C. The cells were harvested by centrifugation at 3,500×g for 30 min followed by suspension in buffer A (20 mM Hepes pH 8, 500 mM NaCl, 1 mM PMSF, 1 mM benzamidine, 1tb Protease inhibitor, 1 mM MgCl_2_, 0.1 mg/ml lysozyme, 0.05 mg/ml DNase). The cells were then broken with a French press and centrifuged at 20,000 g for 15 min. The lysate supernatants were collected and applied to a column packed with chitin resin, bed volume 10 ml (New England Biolabs, Inc.) equilibrated in buffer A. Unbound protein was washed from the column with 10 column volumes of buffer B (20 mM Hepes pH 7, 10% glycerol, 1 mM PMSF, 1 mM benzamidine, 500 mM NaCl). Thiol reagent-induced cleavage was initiated by rapidly equilibrating the chitin resin in buffer B including 0.5 mM dithiothreitol (DTT). The cleavage reaction proceeded overnight at 4°C, after which the protein was eluted from the column. Proteins were dialyzed against buffer C (25 mM Hepes pH 7.5, 10% glycerol, 0.5 mM DTT, 100 mM NaCl) and loaded onto a Q sepharose column equilibrated in buffer C. Unbound protein was washed from the column with 10 column volumes of buffer C and Drp1 was eluted by increasing NaCl concentration of running buffer to 500 mM. Fractions containing Drp1 where then loaded onto a Sephadex S200 column equilibrated in buffer D (25 mM Hepes pH 7.4, 10% glycerol 0.5 mM DTT, 150 mM NaCl). Drp1 containing fractions were mixed and dialyzed with buffer E (25 mM Hepes pH 7.4, 10% glycerol, 0.5 mM DTT, 150 mM KCl).

### Liposome Preparation

Lipid mixtures as indicated were co-dissolved in chloroform:methanol (2∶1), and organic solvents were removed by evaporation under nitrogen stream followed by incubation under vacuum for 2 h. Dry lipid films were resuspended in KCl buffer (150 mM KCl, 0.5 mM EGTA, 10 mM HEPES-KOH pH 7.4). Large Unilamellar Vesicles (LUVs) were formed by the method of Mayer *et al*. [47] using 10 freeze/thaw cycles and unless otherwise stated, two polycarbonate membranes of 0.2 µm pore size for extrusion (Nucleopore, San Diego CA). Lipid concentration was determined by the method of Bartlett *et al*. [48].

### Sucrose gradient centrifugation of liposomes

Purified Drp1 (1 µM) was incubated with 200 µM of liposomes (containing 0.3% rhodamine-PE for detection) for 30 min, at 37°C in a volume of 150 µl (KCl buffer). The protein/lipid mix was diluted to 300 µl in KCl buffer containing 2.4 M sucrose. Then reaction mix was transferred to a centrifuge tube. The 2.4 M sucrose layer was overlaid with 400 µl KCl buffer containing 0.8 M sucrose and 300 µl of KCl buffer containing 0.5 M sucrose. Sucrose step gradients were centrifuged in a TLA-120.2 rotor at 400,000 g for 3 h at 4°C. Four 250-µl fractions were pipetted from the bottom (fraction 4). The top fraction (fraction 1) containing the liposomes as determined by measuring the rhodamine fluorescence, and the bottom fraction (fraction 4) containing the unbound protein, were analyzed by SDS-PAGE and Western blotting.

### Preparation of cytosolic extract

Murine embryonic fibroblasts (MEFs) were resuspended in MB buffer (210 mM mannitol, 70 mM sucrose, 1 mM EDTA, 10 mM Hepes pH 7.4), broken by at least 10 passages through a 25 G1 0.5×25 needle fitted to a 5-ml syringe and centrifuged at 100,000 g for 30 min at 4°C.

### Protein-Lipid Overlay Assay

Membrane lipid-strips were purchased from Echelon Biosciences (Salt Lake City, UT). Strips were used as suggested by the manufacturers. Alternatively, lipid-strips were produced using stock solutions of different phospholipids: lipids were solubilized in 2∶1∶0.8 MeOH:CHCl_3_:H_2_O, spotted onto Hybond C Nitrocellulose and allowed to dry. The nitrocellulose was then blocked with 1% fat-free milk in PBS for 1 hour and incubated for 1 further hour with Drp1 in PBS at 37°C. The strips were washed 3 times with PBS and soaked in anti-Drp1 antibody at 1∶2,000 dilution for 1 hour. The strips were washed twice with PBS and incubated in horseradish peroxidase-conjugated anti-mouse Ab. at a dilution of 1∶5,000. After 3 washes the protein was detected by chemiluminiscence.

### SPR Analyses

SPR measurements were done with a BIAcore X-100 system using a L1 sensor chip (BIAcore). To prepare the L1 chip with liposomes, 10 µl of 20 mM CHAPS were injected at a flow rate of 10 µl/min. Liposomes (2 mM final lipid concentration) were then injected at the same flow rate for 3 min. All injections were performed at 37°C. The L1 chip was regenerated and stripped of liposomes by repeated injections of 20 mM CHAPS and 0.4% SDS until the starting resonance units (RU) reading was obtained. Protein stocks were dialyzed against the same buffer as used for preparing samples for SPR analysis. The protein was passed over the liposome-coated chip at flow-rate of 10 µl/min. The RU change due to buffer was subtracted from the sample signal. Unless otherwise stated, Drp1 concentration was 0.6 µM. Statistical significance was assessed using Student's t-test (unpaired, two-tailed).

### Monolayer Surface Pressure Measurements

Surface pressure measurements were carried out with the MicroTrough-S system from Kibron (Helsinki, Finland) with constant stirring at 37°C. Lipid monolayers were prepared with the following lipid compositions: 80PC/20PE, 28PC/20PE/52PG, 28PC/20PE/52PS, and 54PC/20PE/26CL (mol/mol). The lipid dissolved in chloroform was spread over the surface of 1 ml KCl buffer and the surface area was kept constant. The desired initial surface pressure was attained by adjusting the amount of lipid applied to the air-water interface. After incubation for 10 min to allow the solvents to evaporate, 1 µM Drp1 was injected through a hole connected to the sub-phase, and the change in surface pressure was recorded as a function of time until a stable signal was obtained. Under these conditions, injection of Drp1 in the absence of a lipid monolayer gave a maximal surface pressure below that of the initial surface pressures of the lipid monolayers examined.

### Construction of expression plasmids for WT and Drp1 mutants

Isoform 1, 2 and 3 constructs of human Drp1 were made by PCR amplification of the Drp1 ORFs cloned on pET29a (kindly provided by Dr. C. Blackstone, National Institute of Neurological Disorders and Stroke, Bethesda, USA) using specific primers (5′-GGA ATT C CAT ATG GAG GCG CTA ATT CCT GTC-3′ and 5′-GAA GGC TCT TCC GCA CCA AAG ATG AGT CTC CCG GA-3′) followed by ligation of the restriction enzyme digested PCR product (NdeI/SapI) into prelinearized pTYB1. Site directed mutagenesis was performed using a QuickChange Site-Directed Mutagenesis kit (Stratagene) according to manufacturer's instructions. The primers used for site directed mutations were: Drp1 K557-560A (5′-CTG GAG AGG AAT GCT GGC AAC TTC AGC AGC TGA AGA GTT ATT AGC-3′ and 5′-GCT AAT AAC TCT TCA GCT GCT GAA GTT GCC AGC ATT CCT CTC CAG-3′) Drp1 K569-571A (5′-AGT TAT TAG CAG AAG AAG CAT CAG CAC CCA TTC CAA TTA T-3′ and 5′-ATA ATT GGA ATG GGT GCT GAT GCT TCT TCT GCT AAT AAC T-3′). Drp1 K557-560-569-571A the two pair of primers mentioned above were used sequentially. The constructs were sequenced to verify the mutations.

### GTPase Activity Assay

The GTPase activity of Drp1 was assayed using the malachite green colorimetric phosphate assay as described previously [49]. Briefly, 0.5 µM Drp1 was added to 1 mM GTP and incubated for 30 min at 30°C in 100 µl assay buffer (125 mM KCl, 10 mM Hepes pH 7.4, 1 mM GTP, 4 mM MgCl_2_). 10 µl of the solution was then added to 90 µl Malachite Green stock solution (500 µM Malachite Green, Sigma, 10 mM ammonium molybdate in 1N HCl) in microtiter wells and the absorbance at 650 nm was determined using a microplate reader. For the steady-state kinetic analyses, assays were performed at 30°C in reactions containing 0.5 µM Drp1 and variable GTP concentrations (0, 125, 250, 500, 100 and 2000 µM). Fixes volumes were removed each 5 min for 60 min, and absorbance was measured at 650 nm. The maximal rate of hydrolysis (V_max_) and the substrate concentration at which velocity is one half-maximal (K_0.5_) were calculated in SigmaPlot using nonlinear regression curve fitting. Assays were performed in triplicate and each experiment was repeated three times. Statistical significance was assessed using Student's t-test (unpaired, two-tailed).

### Far-UV Circular Dichroism (CD) Measurements

CD spectra were recorded at 37°C on a Jasco J-810 spectropolarimeter (Jasco Spectroscopic Co. Ltd., Hachioji City, Japan) equipped with a Jasco PTC-423S temperature control unit using a 1-mm path length cell. Data were collected every 0.2 nm at 50 nm/min from 260 to 198 nm with a bandwidth of 2 nm, and results were averaged from 20 scans. All samples were allowed to equilibrate for 30 min before measurements were made. Drp1 (1 µM) was mixed with liposomes (1 mM) in KCl buffer. The contribution of liposomes and buffer to the measured ellipticity was subtracted from all experimental values. Molar ellipticity values were calculated using the expression {θ} = ε/10cnl, where ε is the ellipticity (millidegrees), c is the protein concentration (M), l is the cuvette path length, and n is the number of peptide bond in the protein. To assess the thermal stability of Drp1 sample components were incubated for 30 min in KCl buffer at 37°C prior to beginning of measurements.

### Size exclusion chromatography

Drp1 (1 µM) was mixed with liposomes (1 mM) in KCl buffer and incubated for 30 min at 37°C. Liposomes were then lysed in 2% CHAPS and the insoluble material was removed by centrifugation (100,000 g for 30 min). The supernatant was loaded on Sephadex S200 (Amersham Pharmacia) equilibrated with 25 mM Hepes, 200 mM NaCl, pH 7.5 1% CHAPS, 2 mM DTT. Chromatography was performed using 60 ml buffer and 1 ml fractions were collected. The distribution of Drp1 was analyzed by immunoblotting.

## Results and Discussion

### Drp1 interacts specifically with CL

CL is the signature lipid of mitochondria and constitutes almost 10% by weight of the total lipid content of the organelle [50]. Although it is mainly present in the mitochondrial inner membrane, CL is also found in the MOM, where its concentration is thought to be maximal at membrane contact sites [44]. In addition, it has been found that some physiological events link with an increase in mitochondrial fission, such as apoptosis [51] or mitophagy [52], trigger CL externalization to MOM. We previously reported that Drp1 binds to CL-containing liposomes [21]. Interestingly, different lines of evidence indicate that two other mitochondrial dynamin-like proteins, Mgm1 (in yeast) and Opa1 (in mammals) also interact directly with CL, and that this interaction is important for the functional regulation of these proteins [39]–[41]. Based on these observations, we decided to analyze Drp1:CL interaction in more detail.

**Figure 1:**
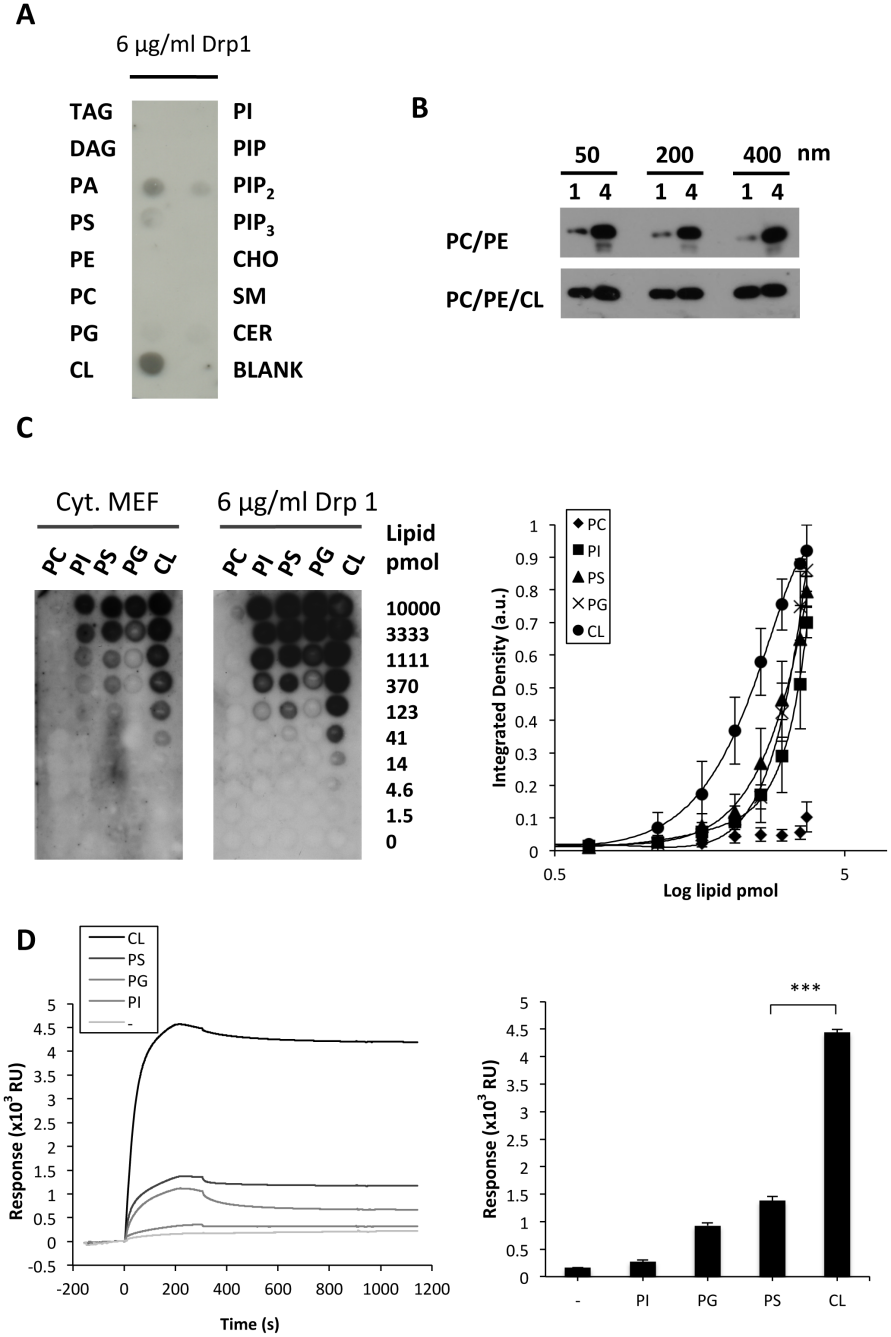
Drp1 interacts with anionic lipids. A) A representative lipid:protein dot-blot showing Drp1 binding. Nitrocellulose membranes spotted with common glycerolipids and sphingolipids present in mammalian cells were incubated with 6 µg/ml Drp1 (Isoform-3). TAG, tryacylglycerol; DAG, diacylglicerol; PA, phosphatidic acid; PS, phosphatidylserine; PE, phosphatidylethanolamine; PC, phosphatidylcholine; PG, phosphatidylglycerol; CL, cardiolipin; PI, phosphatidylinositol; PIP, phosphatidylinositol phosphate; PIP_2_, phosphatidylinositol biphosphate; PIP_3_, phosphatodylinositol triphosphate; CHO, cholesterol; SM, sphingomyelin; CER, ceramide. B) Drp1 partition to liposomes of different sizes after flotation sucrose density gradient centrifugation. Drp1 (1 µg) was incubated at 37°C for 30 min with 200 µM liposomes. Protein/lipid mix (150 µl) was diluted to 300 µl in 2.4 M sucrose containing buffer and transferred to a centrifuge tube. The 2.4 M sucrose layer was overlayed with 400 µl of 0.8 M sucrose followed by 300 µl of 0.5 M sucrose. After ultracentrifugation, four 250 µl fractions were pipetted from the bottom (fraction number 4). Fraction 1 containing the liposomes and fraction 4 containing unbound protein were analyzed by SDS-PAGE and immunoblotting using anti-Drp1 monoclonal antibody. C) Quantitative analysis of Drp1 binding to different phospholipids as determined by protein-lipid overlays. Increasing amounts of the indicated lipids were spotted onto nitrocellulose membranes and binding either cytosol extracted from MEF cells (Cyt. MEF) or recombinant Drp1 (6 µg/ml) was performed. Drp1 immunoreactivity was measured for each spot, normalized for each lipid, and the data were fitted to a sigmoidal dose-response nonlinear regression model using Sigma Plot software. EC_50_ values for CL, PS, PG and PI were 217 pmol, 1091 pmol, 2259 pmol and 3917 pmol. Mean values ±SEM are shown for three independent experiments. D) Surface plasmon resonance analyses of the interaction between Drp1 (0.6 µM) and LUVs containing the indicated lipids immobilized on the surface of L1 sensor chip. Left panel: representative sensorgrams showing binding and dissociation kinetics. Right panel: summary of binding responses obtained in 3 to 5 independent experiments. Liposome lipid compositions (mol/mol) were as follows: 80PC/20PE (-), 28PC/20PE/52PI (PI), 28PC/20PE/52PG (PG), 28PC/20PE/52PS (PS), and 54PC/20PE/26CL (CL). Values are means of more than four independent experiments ±SEM. Drp1 binding to CL containing liposomes differed from PS containing liposomes with ***: p<0.001.

First, we performed a lipid dot-blot analysis in which recombinant Drp1 was incubated with a nitrocellulose membrane containing some of the most common glycerolipids and sphingolipids present in mammalian cells. Among the lipid species examined, Drp1 bound most strongly to CL (Fig. 1 A), less strongly to the anionic lipids phosphatidylserine (PS) and phosphatidic acid (PA) and interestingly not detectably to the polyanionic lipid phosphatidylinositol (3,4,5)-trisphosphate (PIP_3_) even though this lipid displays higher net negative charge than CL. Dose-response experiments indicated that recombinant Drp1 bound to CL with ∼5-fold, ∼10-fold and ∼18-fold higher potency than to PS, PG and PI, respectively (Fig. 1C). Importantly, additional lipid dot-blot assays revealed that the endogenous Drp1 from MEF cells also recognized a variety of anionic phospholipid species and also displayed a similar preference for CL (Fig. 1C). Considering that phospholipids spread on a nitrocellulose membrane do not form a lipid bilayer, an extrapolation from these data would suggest that the Drp1:CL interaction does not rely on CL-mediated changes in the physical properties of the bilayer, such as induction of lateral segregation of membrane lipids into domains or generation of negative membrane monolayer curvature stress. To further address this point, we examined the interaction of Drp1 with PC/PE and PC/PE/CL vesicles of different sizes ranging from 50 nm to 400 nm. We observed almost no binding of Drp1 to PC/PE vesicles, while a similar binding was detected for CL containing vesicles, independently of their size (Fig. 1B). These results support the notion that Drp1 interaction with CL-containing liposomes is not affected by the membrane intrinsic net curvature.

To study the lipid-binding properties of Drp1 in real-time, we performed surface plasmon resonance studies (SPR). To this end, LUVs with different lipid compositions were immobilized on the surface of the L1 sensor chip and used as ligands to probe the kinetics of Drp1 binding. Representative SPR sensorgrams are shown in Figure 1D. Maximal binding was observed with LUVs containing CL, while Drp1 did not bind to LUVs composed of PC/PE possessing zero net negative charge. Interestingly, replacing CL with other anionic lipids (PS, PI, PG) while maintaining the same net negative charge on the vesicle, did not reproduce the strong binding response observed with CL-containing vesicles, supporting the notion that the Drp1:CL interaction does not rely exclusively on electrostatic forces.

**Figure 2:**
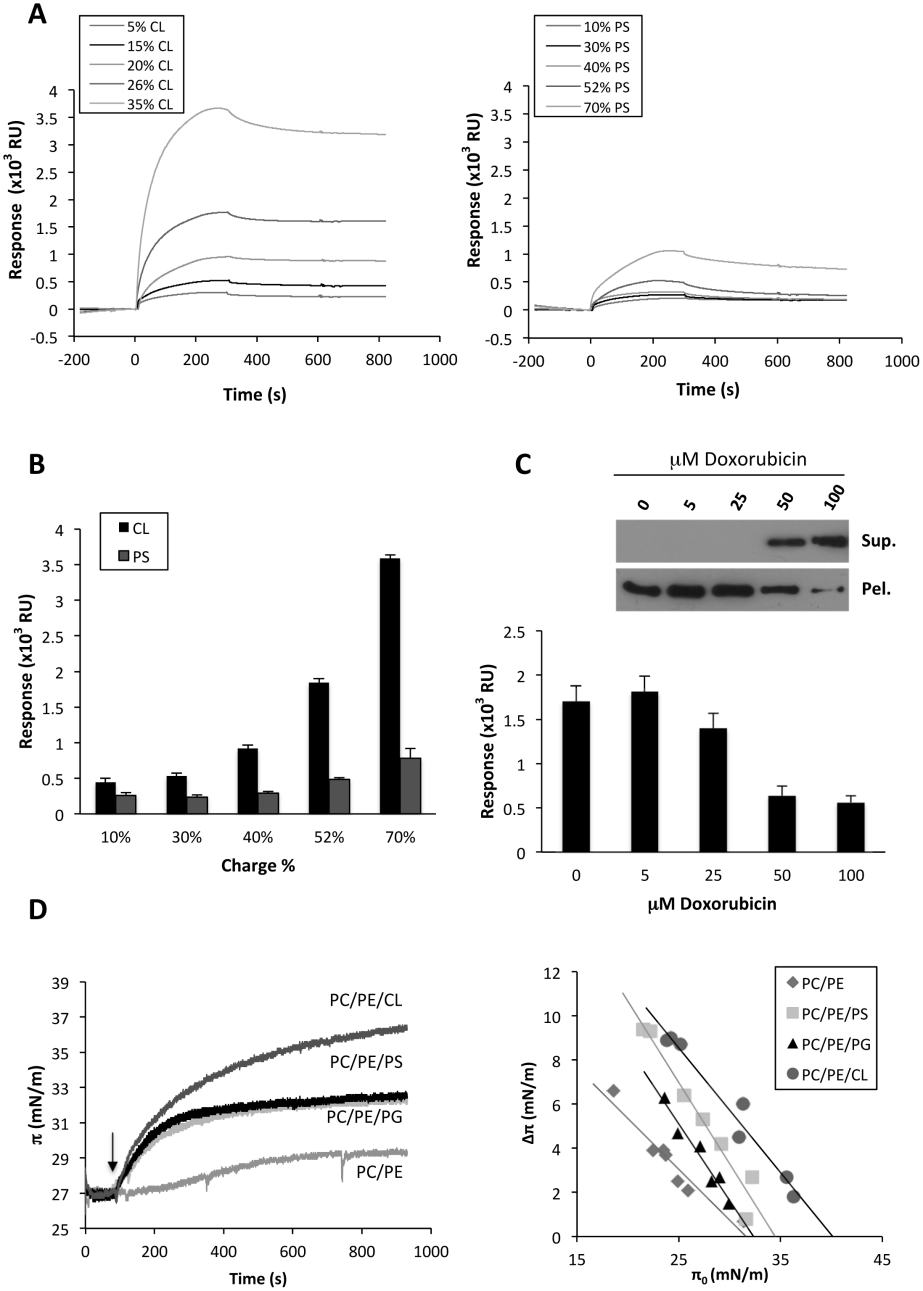
Drp1 binds preferentially to CL compared to other anionic lipids. A) Representative sensorgrams depicting interaction of Drp1 (0.3 µM) with LUVs containing increasing concentrations of either CL or PS. Liposome lipid compositions were as follows: 75PC/20PE/5CL (5% CL), 65PC/20PE/15CL (15% CL), 60PC/20PE/20CL (20% CL), 54PC/20PE/26CL (26% CL), 45PC/20PE/35CL (35% CL) and 70PC/20PE/10PS (10% PS), 50PC/20PE/30PS (30% PS), 40PC/20PE/40PS (40% PS), 28PC/20PE/52PS (52% PS), 10PC/20PE/70PS (70% PS). B) Summary of Drp1 (0.3 µM) binding responses obtained for CL and PS containing liposomes. Each bar represents the average of at least three independent experiments±SEM. C) Upper panel: Drp1 (1 µM) was incubated with freeze/thawed liposomes composed of 54PC/20PE/26CL (mol/mol) in the presence of the indicated concentrations of the CL-interacting drug doxorubicin. Liposomes were sedimented by centrifugation, and the pellet (Pel.) and supernatant (Sup.) fractions were analyzed for the presence of Drp1 by Western blotting. The blot is representative of three independent experiments. Lower panel: binding responses obtained for Drp1 (0.3 µM) in the presence of increasing concentrations of doxorubicin. Liposome lipid composition was 54PC/20PE/26CL (mol/mol). Mean values ±SEM are shown for three independent experiments. D) Left panel: changes in surface pressure (π) of lipid monolayers of 80PC/20PE, 28PC/20PE/52PS, 28PC/20PE/52PG or 54PC/20PE/26CL after addition of Drp1 to the subphase were measured as a function of time. Arrow corresponds to time of protein addition. Right panel: changes in surface pressure (Δπ) of lipid monolayers were measured as a function of the initial surface pressure (π_0_). The data are fitted to a straight line, and the intercepts with the x-axis define the monolayer exclusion pressure (π_c_), which is a measure of the membrane penetrability. The values of π_c_ are 31.61 mN/m for PC/PE, 32.25 mN/m for PC/PE/PG, 34.45 mN/m for PC/PE/PS and 40.12 mN/m for PC/PE/CL.

Next, we analyzed Drp1 binding to vesicles containing increasing amounts of CL and PS. At all concentrations tested, Drp1 binding responses were lower for PS-containing liposomes relative to CL-containing liposomes (Fig. 2A, 2B). Unfortunately kinetic rate constants could not be obtained by fitting sensorgrams to a first-order binding model. Similar observations were previously reported for other protein:membrane interactions analyzed by SPR [53]–[55]. To investigate further the specificity of Drp1 interaction with CL, we examined the effect of the CL-interacting drug doxorubicin. Drp1 was incubated with CL-containing liposomes in the presence of increasing amounts of doxorubicin and the lipid-containing and lipid-free fractions were separated by centrifugation. The amounts of liposome-bound Drp1 (pellet, Pel.) and free Drp1 (supernatant, Sup.) were analyzed by SDS-PAGE followed by immunoblotting (Fig. 2C, upper panel). These data clearly show that increasing concentrations of doxorubicin led to displacement of Drp1 from the CL-containing liposomes, and this effect was confirmed by SPR experiments which also showed that doxorubicin efficiently competes for Drp1 binding to liposomes (Fig. 2C, lower panel).

Next, we analyzed the interaction of Drp1 with lipid monolayers using a Langmuir balance. Lipid monolayers composed of 80PC/20PE, 28PC/20PE/52PG, 28PC/20PE/52PS or 54PC/20PE/26CL were prepared with constant surface area and an initial pressure of 28 mN•m^−1^. Recombinant Drp1 was added to the subphase, and the resulting increase in monolayer surface pressure was monitored in real-time. As shown in Figure 2D, the surface pressure of the monolayers containing PS, PG or CL started to rise immediately after injection of Drp1, while a pronounced lag time was observed in the case of the electrically-neutral PC/PE monolayers. These results indicate that Drp1 association with lipid monolayers depends at least in part on an electrostatic component. It is also notable that Drp1 causes a much larger surface pressure increase in CL-containing monolayers as compared to PS- or PG-containing monolayers even though the net electrical charge is the same in all cases (Fig. 2D). This suggests that other properties of CL may also contribute to the interaction with Drp1. To obtain a quantitative measure of the ability of Drp1 to penetrate into lipid monolayers, critical surface pressure values were determined. In these experiments, the increase in surface pressure (Δπ) upon Drp1 addition was measured as a function of the initial surface pressure (π_0_). The data are fitted to a straight line, and the x-intercepts correspond to the monolayer critical surface pressure (π_c_). The results in Fig 2D show that the penetrating potency of Drp1 was highest for CL-containing monolayers and lowest for PC/PE monolayers, while PG- and PS-containing monolayers displayed intermediate π_c_ values.

To sum up, results obtained using three independent lipid-interaction assays (lipid dot-blot, SPR and lipid monolayer surface pressure measurements) all concur in showing that Drp1 interacts preferentially with membranes containing CL compared to membranes containing other anionic lipids.

### Drp1 B Insert mediates interaction with CL

In dynamins, the PH domain drives the binding of the protein to phosphoinositide-containing membranes, mediates the clustering of the phosphoinositides, and coordinates the regulation of GTPase activity through its membrane association [56], [57]. The dynamin-related protein, Drp1 does not contain a PH domain and the role of its B insert on protein activity is unclear. Recently, it has been shown that B insert of the yeast protein Dnm1 contains a short motif required for association with the mitochondrial adaptor protein Mdv1 [58]. However, this motif is strictly conserved only among fungi, and moreover, Mdv1 orthologues have not been identified in flies, worms or vertebrates. B insert is predicted to be unstructured and it has also been proposed, that similar to the PH domain, it could constitute a putative membrane interaction site [24], [59]. However, this hypothesis has been challenged since it has been suggested that the function of B insert is to allosterically modulate protein assembly [60].

**Figure 3:**
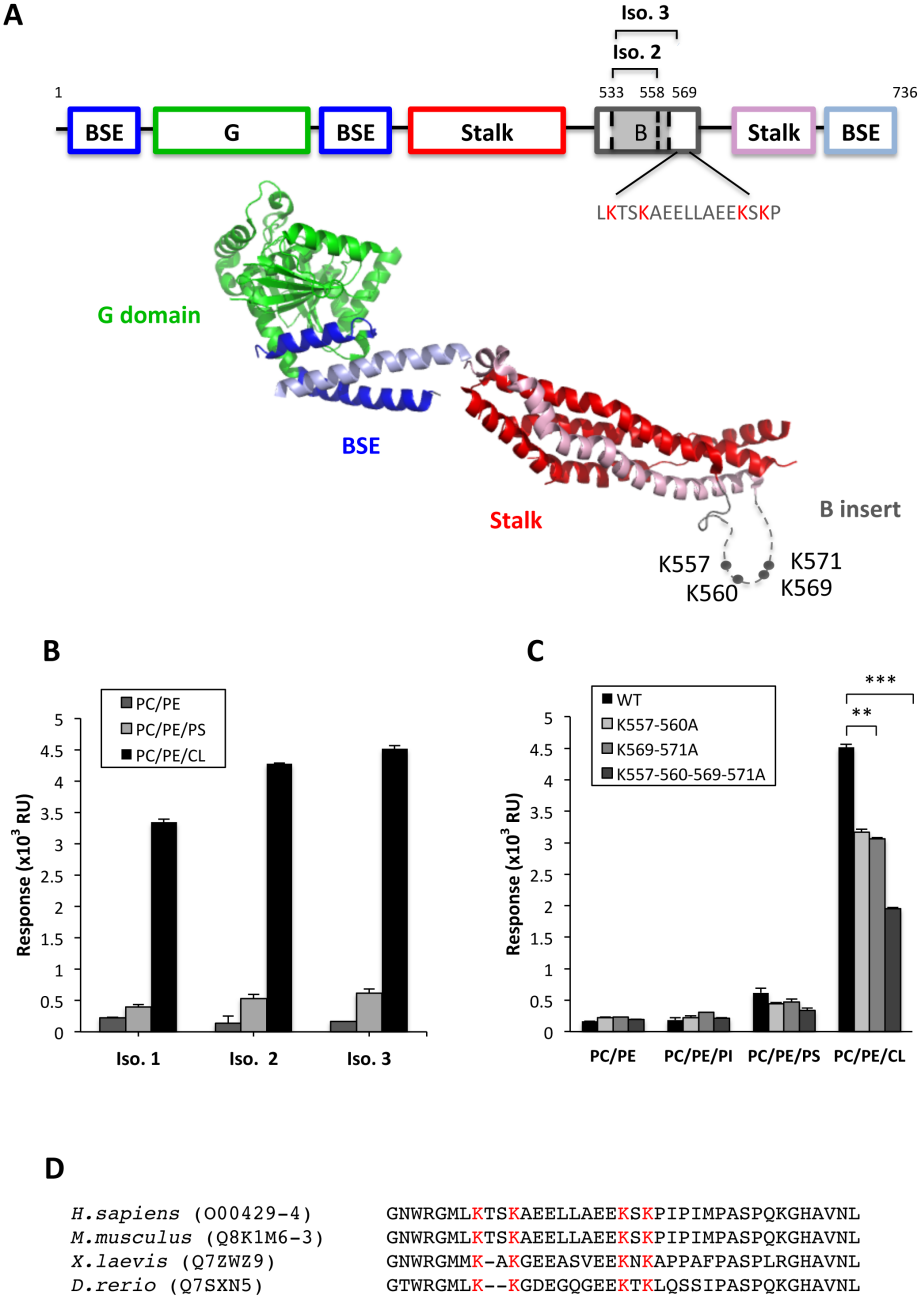
B insert is required for Drp1 interaction with CL. A) Upper panel: schematic representation of the structure-based domain organization of Drp1. Lower panel: Ribbon-type representation of Drp1 isoform 3 illustrating the localization of G domain (green), BSE (blue), B insert (gray) and stalk (red-pink) (Protein Data Bank code 4BEJ). The lysine residues mutated to alanine are represented (grey circles). B) Binding responses obtained by surface plasmon resonance spectroscopy for the three splice variants of Drp1 (each at 0.6 µM) in 80PC/20PE, 28PC/20PE/52PS and 54PC/20PE/26CL (mol/mol). Mean values ±SEM are shown based on three independent experiments. C) Binding responses obtained for the Isoform-3 WT, and the Isoform-3 B insert lysine mutants, K557-560A, K569-571A and K557-560-569-571A. Liposome lipid composition was 80PC/20PE (−), 28PC/20PE/52PI (PI), 28PC/20PE/52PS (PS), 28PC/20PE/52PG (PG), and 54PC/20PE/26CL (CL) (mol/mol). Mean values ±SEM are shown for three independent experiments with **p<0.01, ***p<0.001. D) Amino acid sequence alignment of a B insert segment comprehending residues 550–588 of human Drp1 Isoform-3 with Drp1 from *M. musculus*, *X. laevis*, and *D. rerio*, using Clustal Omega with manual adjustment. Lysines responsible of Drp1:CL interaction are highlighted in red.

To study the role of B insert in the Drp1:CL interaction, we made three naturally occurring splice variants of human Drp1 that differ in the length of the B insert as follows: Variant 1, the full-length 736-residue protein; Variant 2 lacking 26 residues in B insert; and Variant 3 lacking 37 residues in B Insert (Fig. 3A). The three isoforms were purified without affinity tags. All three isoforms eluted as a mixture of dimers and tetramers after gel filtration and showed virtually no contaminating proteins (data not shown).

We first assessed the lipid-binding pattern of the three isoforms by SPR. For this purpose, liposomes composed of 80PC/20PE, 28PC/20PE/52PS and 54PC/20PE/26CL were prepared. All three isoforms interacted preferentially with anionic phospholipids, although they all showed markedly higher affinity for CL-containing liposomes. Of the 3 variants, isoform 3 showed slightly higher capacity for binding CL followed by variant 2 and variant 1 (Fig. 3B).

The B insert region in all isoforms of Drp1 contains two pairs of lysine residues (K557/K560 and K569/K571 for variant 3) that can be SUMOylated, a process which has been linked to its activity cycle [28]. Indeed, B insert of isoform 3 contains essentially only the lysine-containing module (Fig. 3A). Since all three isoforms showed a similar capacity to bind CL we set up to test whether the ability of B insert to interact with CL was dependent on the lysines known to be SUMOylated. To test this we first replaced all four lysines in isoform 3 with alanine residues (4KA mutant) and analysed the binding capacity of this mutant to bind different lipids. As shown by SPR (Fig. 3C) the 4KA mutant displayed significantly impaired capacity for binding to CL-containing membranes. We next examined the contribution of the two different pairs of residues by reintroducing the lysines two by two in the context of the 4KA mutant. Reintroduction of either K557/560 or K569/571 both restored partially Drp1 binding to CL, thus suggesting that all four lysines are implicated in the Drp1:CL interaction (Fig. 3C). Amino acid sequence alignment of the B insert done using Clustal Omega [61], shows that vertebrates share the four lysines responsible for Drp1:CL interaction (Fig. 3D).

To sum up, the results above, together with the fact that B insert of Drp1 is localized at the equivalent position as the PH domain in dynamins and the CL-interacting residues of Opa1 and Mgm1 [39], [41], strongly suggest that B insert represents the CL-interacting domain of Drp1.

### Interaction with CL enhances Drp1 GTPase activity

**Figure 4:**
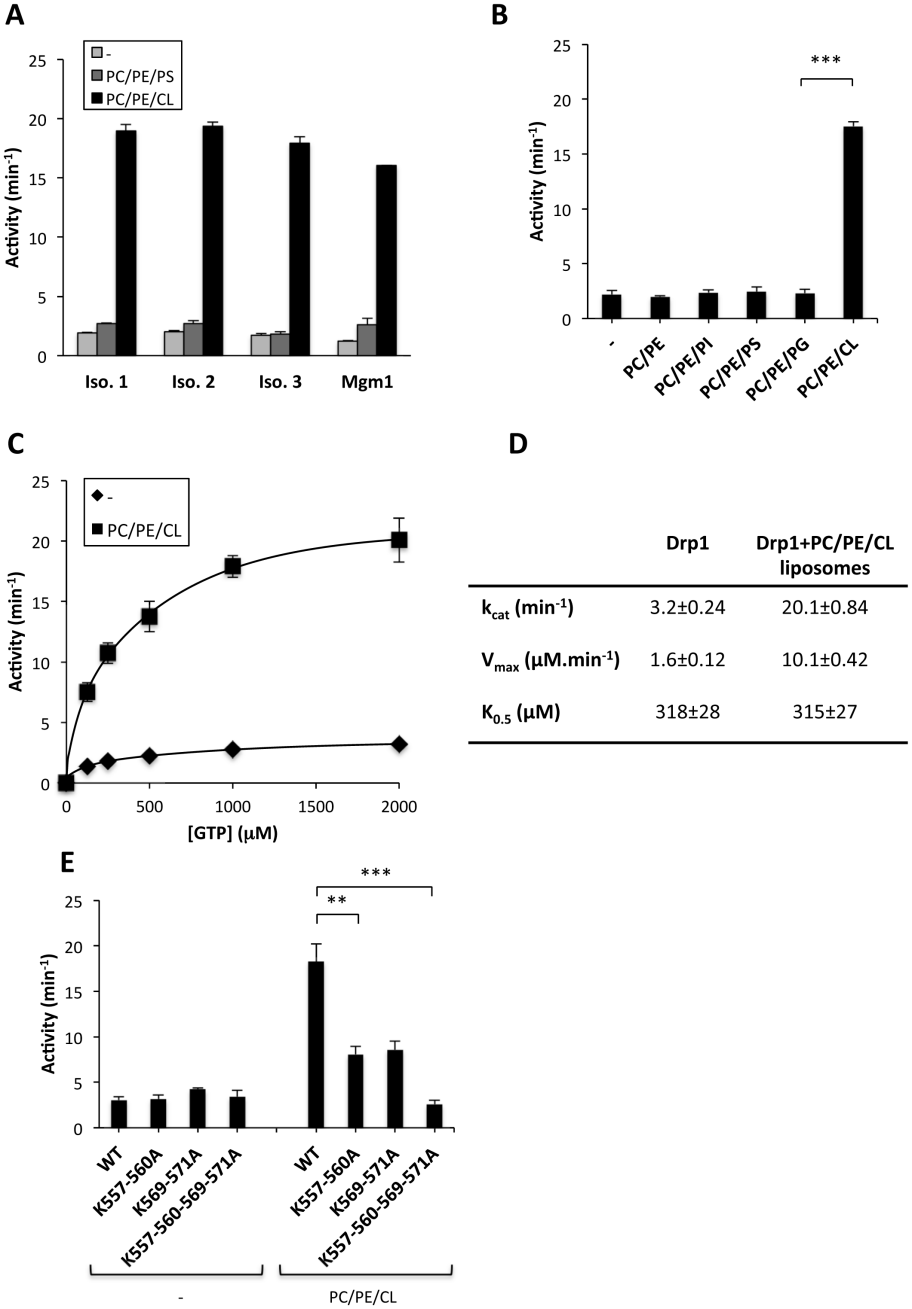
CL stimulates Drp1 GTPase activity. A) GTPase activity of Drp1 Isoforms 1, 2 and 3, and Mgm1 (0.5 µM each) in solution and in presence of 80PC/20PE, 28PC/20PE/52PS or 54PC/20PE/26CL (mol/mol) (0.5 mM) liposomes. Values are means of three independent experiments ± SEM. B) GTP hydrolysis activity of Drp1 Isoform-3. Drp1 GTPase activity in 54PC/20PE/26CL (mol/mol) differed from 28PC/20PE/52PG (mol/mol) with ***: p<0.001. Mean values ±SEM are shown for three to seven independent experiments. C) Steady-state kinetics of Drp1 (0.5 µM) GTP hydrolysis measured in absence or presence of 54PC/20PE/26CL (mol/mol) liposomes. Values are means of three to five independent experiments ±SEM. D) Table showing Drp1 kinetic parameters determined as described in *Material and Methods*. Turnover number (k_cat_), maximum reaction rate (V_max_), and substrate concentration that gives half maximal velocity (K_0.5_). Mean values ±SEM are shown for four to six independent experiments. E) GTP hydrolysis activity of Drp1 Isoform 3 WT protein and the Drp1 Isoform 3 carrying lysine mutations in B insert. Reactions were performed in solution in the absence (−) or presence of CL-containing vesicles (PC/PE/CL). Values are means of three independent experiments ±SEM with **p<0.01, ***p<0.001.

**Figure 5:**
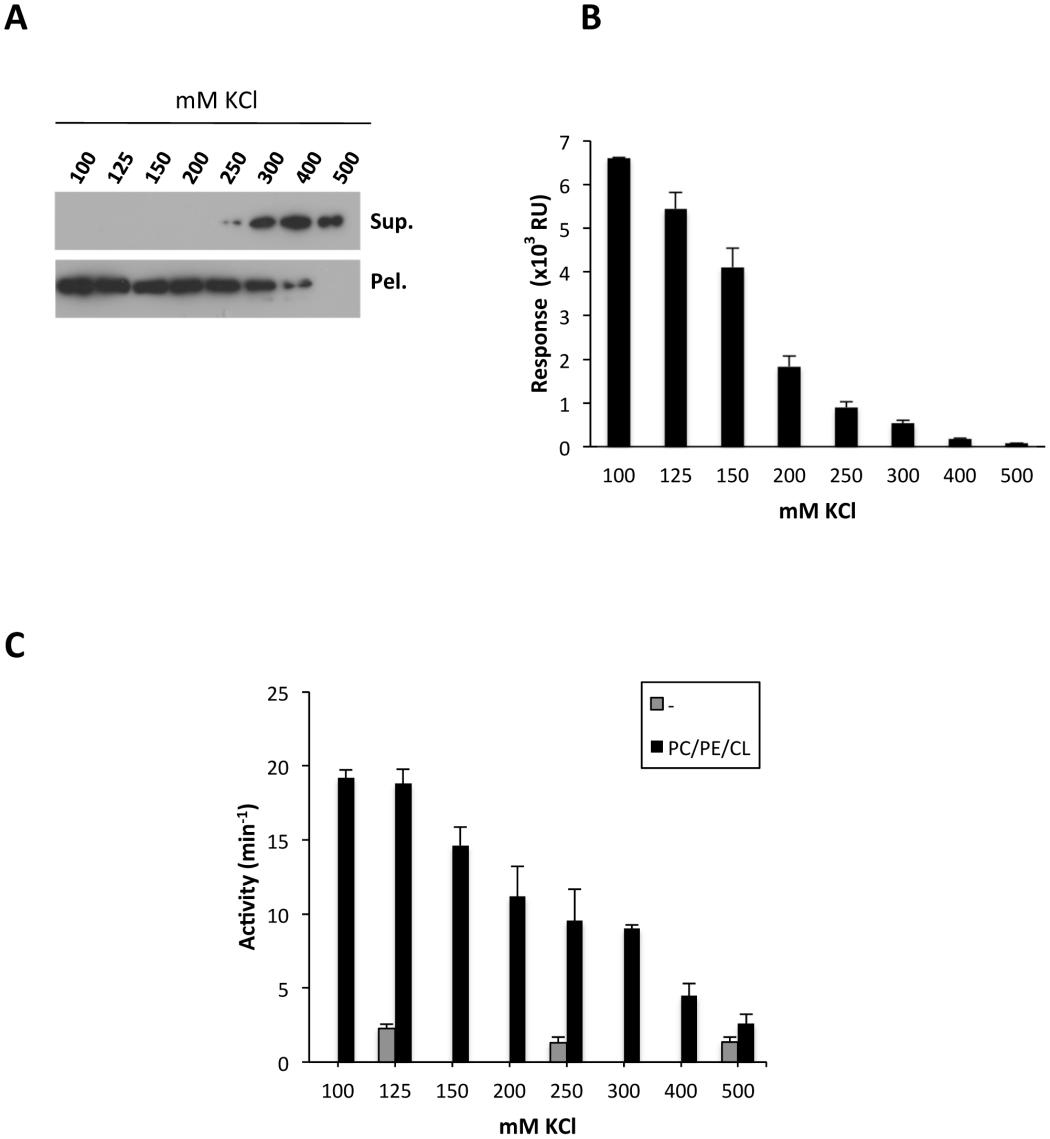
Ionic strength increase affects Drp1 interaction with CL. A) Lipid cosedimentation assay. Drp1 (1 µM) was incubated for 30 min with freeze/thawed liposomes composed of 54PC/20PE/26CL (mol/mol) in the presence of increasing amounts of KCl, followed by sedimentation of the liposomes by low speed centrifugation, and immunoblot analysis of Drp1 contents in the liposome-free (Sup.) and liposome-containing (Pel.) fractions using anti-Drp1 monoclonal antibody. B) Surface Plasmon Resonance analyses of Drp1 (0.6 µM) interaction with 54PC/20PE/26CL (mol/mol) liposomes in presence of increasing amounts of KCl. Each bar represents the average of at least three independent experiments±SEM. (C) GTP hydrolysis activity of Drp1 Isoform-3. Drp1 GTPase activity was measured in solution and in presence of 54PC/20PE/26CL (mol/mol) LUVs increasing the ionic strength of the buffer. Values are means of three independent experiments ±SEM.

It has been shown that the GTPase activities of dynamin, Opa1 and Mgm1 short isoform all increase when the proteins assemble into high order oligomers in membranes containing polyanionic lipids [38], [39], [41], [56], [57], [62]. We therefore examined the effect of different phospholipids on Drp1 GTPase activity. Incubation of Drp1 with CL-containing liposomes stimulated GTP hydrolysis by all three splice variants described above to almost the same degree (Fig. 4A). CL also stimulated the GTPase activity of Mgm1 (Fig. 4A), which is in agreement with previously reported data [40], [41]. In contrast, vesicles containing PS while maintaining the same net charge did not enhance the low basal GTPase activity of any of the three isoforms (Fig. 4A). To characterize the effect of anionic phospholipids further, we prepared liposomes composed of PC/PE plus variously PI, PS, PG or CL as described in the legend to Fig. 4. Of the lipids tested, we found that only CL increased the rate of GTP hydrolysis (Fig. 4B, 4C and 4D). Frölich C. *et al*. [24] have previously shown that the low basal GTP hydrolysis rate of Drp1 can be stimulated 10-fold in presence of 100% PS liposomes. We have not tested whether 100% PS-containing liposomes can stimulate the Drp1 GTPase activity. What we report here is that at more physiological concentrations, CL is more efficient than PS in triggering Drp1 activity. However, we do not exclude the possibility that under some experimental conditions, such as low MgCl_2_ concentration, PS could be a strong activator of Drp1. K_0.5_ values obtained for Drp1 alone and in the presence of CL-containing liposomes were essentially the same (Fig. 4D) thus showing that Drp1 has a similar affinity for GTP in both conditions. However, CL containing liposomes increased the steady-state level of GTP hydrolysis up to 6 fold (Fig. 4C and 4D). It is important to emphasize that although Drp1 can bind to other anionic phospholipids, only CL appeared capable of enhancing Drp1 GTPase activity. To obtain more insight into this phenomenon, we analysed the effect of ionic strength on Drp1 lipid-binding and GTP-hydrolysing activities. Increasing the salt concentration of the buffer gradually reduced Drp1 binding to CL-containing liposomes as shown by the sedimentation assay as well as by SPR (Fig. 5A and 5B). The decrease in Drp1:CL interaction correlated with a reduction of Drp1 GTPase activity (Fig. 5C).

Since the results presented thus far indicate that specific binding of Drp1 to CL is essential to stimulate its GTPase activity, we examined the effect of mutations in B insert on CL-dependent GTP hydrolysis. As shown above (Fig. 3C), the Drp1-4KA mutant had a reduced capacity to interact with CL. Determination of the GTPase activity of this mutant in the presence or absence of CL containing vesicles shows that the reduced affinity for CL correlates perfectly with a reduced capacity of the CL-containing liposomes to stimulate its GTPase activity (Fig. 4E). Importantly, the basal GTPase activity of the 4KA mutant was unaffected. Reintroduction of each of the lysine pairs (K557/560 or K569/571) separately into B insert both led to similar partial restoration of cardiolipin-stimulated GTPase hydrolysis although the values obtained with the WT protein were not reached in either case (Fig. 4E). Based on these data, we conclude that all four lysines present in B insert of Drp1 participate in lipid association and stimulation of GTPase activity. In agreement with our observations, it has recently been seen that addition of anionic lipids containing liposomes to a Drp1 construct lacking the B insert does not increase the GTP hydrolysis rate [24]. In summary, our results strongly suggest that the interaction of Drp1 B insert with CL is intimately linked to Drp1 functional activation. In contrast to our findings, Strack and Cribbs observed that the B insert is dispensable for mitochondrial recruitment, association with Mff and basal and protonophore-stimulated mitochondrial fission [60]. In this regard it is quite possible that the mechanisms of Drp1 recruitment and activation in the MOM vary depending on the cell context. CL dependent Drp1 binding and activation may be relevant in cellular processes where CL has been proposed to be transported from the mitochondrial inner membrane to the MOM such as apoptosis and mitophagy [51], [52].

### Drp1 self-assembles on CL containing liposomes

**Figure 6:**
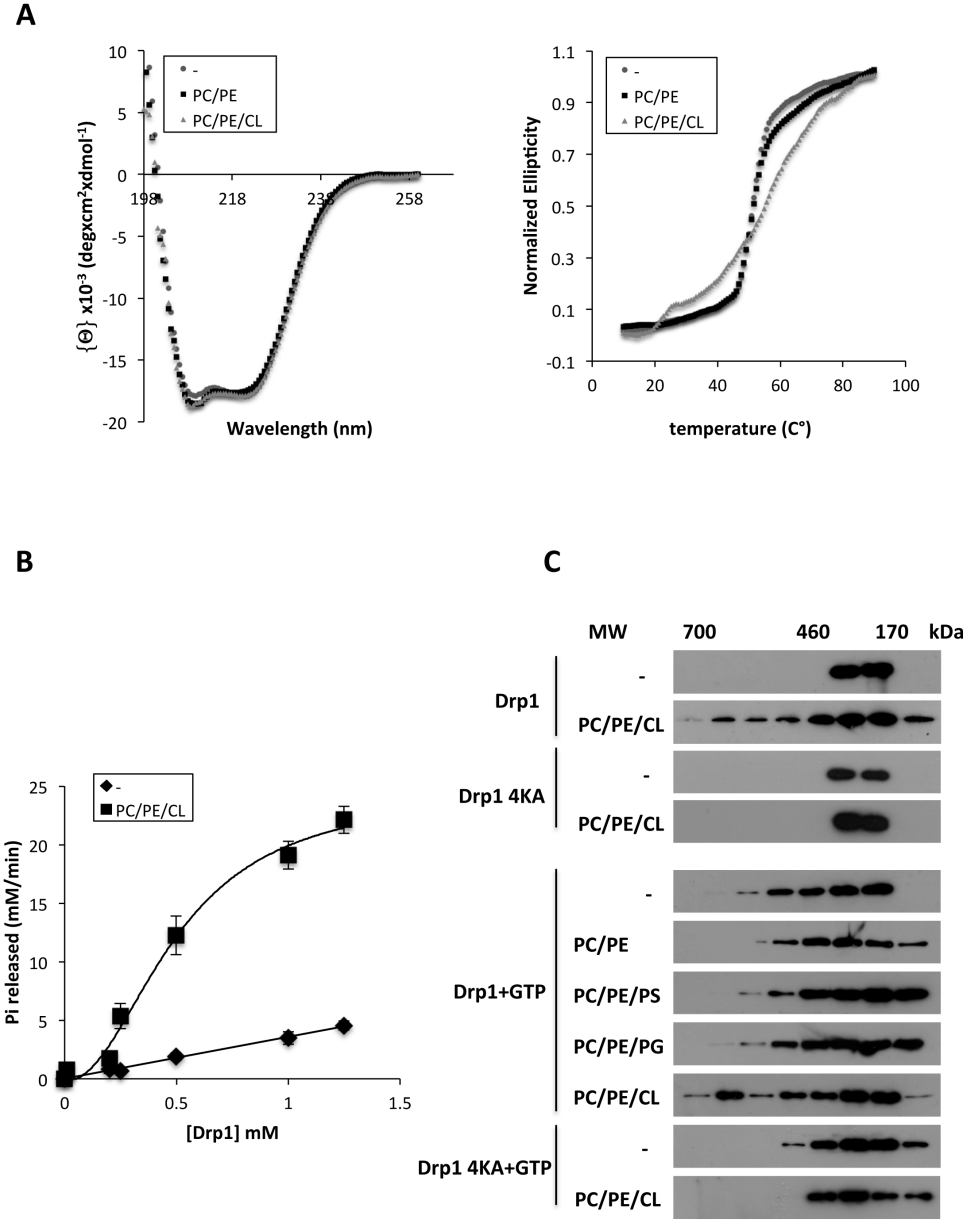
Interaction with CL triggers Drp1 oligomerization. A) Left panel: representative far-UV CD spectrum of Drp1 in the absence (−) or presence of LUVs composed of 80PC/20PE or 54PC/20PE/26CL (mol/mol). Right panel: thermal denaturation curves for Drp1 incubated in the absence (−) or presence of 80PC/20PE or 54PC/20PE/26CL (mol/mol) liposomes monitored by CD at 222 nm. The ellipticity values were normalized to compare Drp1 thermal denaturation rates in the absence and presence of vesicles. Drp1 and SUV concentrations were 1 µM and 1 mM, respectively. B) Rate of GTP hydrolysis was measured by using malachite green colorimetric assay as described under Materials and Methods. Increasing concentrations of Drp1 were incubated without (⧫) or with (▪) 54PC/20PE/26CL (mol/mol) liposomes (400 µM) and 1 mM GTP at 37°C. The GTPase activity of Drp1 in solution is linearly proportional to its concentration; in contrast, in presence of PC/PE/CL liposomes Drp1 exhibits sigmoidal behavior. Values are means of two to four independent experiments ±SEM. C) Gel-filtration analysis of Drp1 WT and 4KA mutant on Sephadex S200. Drp1 (1 µM) was incubated with or without GTP (1 mM) and liposomes (1 mM) in KCl buffer containing 4 mM of MgCl_2_ for 30 min at 37°C. Indicated samples were treated with 2% CHAPS (w/v) and applied to the column equilibrated with KCl buffer+1% CHAPS (w/v). Fractions were collected and analyzed by SDS-PAGE and immunoblotting using anti-Drp1 monoclonal antibody. Liposome lipid composition was: 28PC/20PE/52PI, 28PC/20PE/52PS, 28PC/20PE/52PG, and 54PC/20PE/26CL (CL) (mol/mol).

Classical dynamins are known to undergo stimulated GTPase activity upon lipid-induced self-assembly [37], [63], an event that has also been described for some dynamin-like proteins [40], [41]. Thus, we decided to investigate structural changes occurring in Drp1 upon interaction with CL. Far-UV CD spectra for the protein alone in solution or in the presence of liposomes with or without CL showed minor differences in the secondary structure of the protein (Fig. 6A). Using the program CD-Pro, we calculated that the α-helix and β-strand content were about 52.95±1.7% and 10.79±2.67% for the protein in solution, 55.85±2.17% and 8.57±4.39 for the protein with PC/PE vesicles and 54.31±4.17% and 11.15±4.07% for the protein with PC/PE/CL vesicles respectively. These values correlate well with those obtained by X-ray diffraction [24]. Thus we conclude that Drp1 is correctly folded in all conditions and that the secondary structure is not significantly modified in the presence of vesicles.

In order to study changes of the tertiary or quaternary structure of Drp1 we followed the thermal denaturation profile of the protein by CD at 222 nm. Drp1 in solution showed a sigmoidal melting curve and started to unfold rapidly at temperatures above 50°C. In contrast, addition of CL-containing liposomes reduced the cooperativity of the denaturating process. This suggests that Drp1 is able to adopt different conformations and that interaction with CL leads to an increase in higher order protein stability.

A common theme to emerge from the study of dynamins is that oligomerization plays an important role in regulating their functional state [56], [57]. It had previously been shown that stimulated dynamin GTPase activity was highly cooperative in the presence of PIP_2_ containing vesicles, which reflects higher order dynamin self-assembly [56], [57]. We therefore examined the concentration dependence of Drp1's GTPase activity in solution and in the presence of CL-containing liposomes. Whereas the GTP hydrolysis rates of Drp1 in solution increased linearly with increasing protein concentration, hydrolysis rates increased in a sigmoid fashion in the presence of CL-containing liposomes, reflecting positive cooperativity (Figure 6B). Thus these data suggest that Drp1's GTPase activity may be stimulated by self-assembly in CL-containing vesicles.

We next studied the oligomerization of Drp1 WT and 4KA mutant under various conditions using size exclusion chromatography. The proteins were incubated with or without GTP in the presence or absence of liposomes of various lipid compositions. Lipid vesicles were then solubilized with 2% CHAPS and fractionated by size exclusion chromatrography (Fig. 6C). We found that GTP alone was able to stimulate the formation of Drp1 WT as well as 4KA complexes. In the presence of PC/PE/CL vesicles, Drp1 WT eluted in a size fraction corresponding to about 700 kDa and greater even in the absence of GTP. In contrast, the elution profile of the Drp1 4KA mutant remained unaltered. Addition of PC/PE, PC/PE/PS or PC/PE/PG vesicles did not modify further the elution profile of Drp1 WT. Thus, we conclude that interaction with CL is able to stimulate the assembly of Drp1 into higher MW complexes.

## Conclusions

Cardiolipin has been described as the membrane anchor for a variety of peripheral membrane proteins such as cytochrome c [64], [65], caspase 8 [66] and truncated Bid [67]. In addition to serving as a “membrane-anchor”, interaction with CL may also regulate protein function. In this work, we describe several novel findings supporting a physical and functional interaction between CL and the mitochondrial fission protein Drp1. First, we demonstrate that all three splice variants of Drp1 (isoform 1, 2 and 3) which differ in the length of the B insert, exhibit preferential binding to CL compared to other anionic phospholipids. Moreover, we also found that this is the case not only for recombinant forms of Drp1, but also for endogenous Drp1 isolated from MEF cells. Second, we have found that B insert is responsible for the interaction with CL. Although many proteins interact with CL, specific sequence motifs that enable the interaction have rarely been identified [67]. Proteins belonging to the superfamily of dynamins can be divided into two sub-groups, (i) the dynamins, and (ii) the dynamin-like proteins. In the dynamins, the PH domain located at the tip of the stalk is responsible for the interaction with PIP_2_. In the dynamin-like protein Mgm1, the interaction with CL occurs through a lysine module located also at the tip of the stalk. In Drp1, replacement of lysines 557, 560, 569 and 571 (in isoform 3) within B insert for alanines impairs the capacity of Drp1 to interact with CL. It is worth to mention that these lysine residues are located in a predicted disordered region that could provide the necessary flexibility for docking to CL. Coincidentally, a four lysine module located in an unstructured loop in an equivalent region of the human dynamin-like MxA protein serves as the lipid-binding moiety [68]. Our results demonstrate that several lysines in the B insert are required for the proper lipid interaction of Drp1 and provide a lipid interface similar to the PH domain in the dynamins.

Finally, we identify two different mechanisms by which the specific mitochondrial lipid CL could regulate Drp1 function. First, Drp1 directly interacts with CL leading to assembly of the protein into higher order oligomers; and second, the interaction with CL stimulates Drp1 GTPase activity. During the revision of our manuscript, similar findings were reported by Mcdonald *et al*. (2014) [69]. We speculate that stimulation of GTPase activity is dependent on generation of the higher molecular weight forms characteristic of the lipid bound form although this still needs to be shown directly. Interestingly, it is well established that dynamins have a relatively high basal GTPase activity that increases when the protein self-assembles in PIP_2_ containing membranes. Moreover, in contrast to previous results [70], it has been proposed that the GTPase activity may be altered by mutations in the PH domain [37]. Similar molecular mechanisms have been described for proteins implicated in shaping mitochondria. The dynamin related GTPase Opa1 has a low basal rate of GTP hydrolysis that is enhanced by association with membranes containing anionic phospholipids. As described for dynamins, lipid association triggers protein oligomerization and enhancement of GTP hydrolysis rate. Also in this case, mutations that affects lipid-binding such as Dominant optic atrophy causing Q785R results in defective CL-stimulated GTP hydrolysis [39]. In the case of the yeast ortholog Mgm1, lipid binding through several conserved lysines is required for protein assembly and stimulated GTPase activity [41]. Our data pinpoint the requirement for lipid association of Drp1 and suggest that its functional activation follows a similar molecular mechanism as that described for other members of the dynamin superfamily.

To sum up, we have found that a module of four lysines located in the B insert is essential for Drp1 interaction with CL. Importantly, it has been previously found that these lysines can be SUMOylated, although the impact of SUMOylation on Drp1 function remains controversial. Drp1 SUMOylation has been shown to be promoted by Bax during apoptosis, resulting in the stable association of the protein with mitochondria [71]. On the other hand, the role of this post-translational modification has been proposed to be a way to induce Drp1 oligomers disassembly and probably to inactivate Drp1, in a manner similar to what has been proposed for septins in *S. cerevisiae*, [28], [72]. Consistently, it has been found that SUMO-2/3-specific protease SENP3 mediates Drp1 deSUMOylation facilitating protein localization at mitochondria and promoting fragmentation [29]. In line with the latter results, we propose that upon deSUMOylation by SENP3, Drp1 would be recruited at mitochondria by Mff, MiD49 or MiD51. Once in contact with the membrane, CL would stimulate its self-assembly and GTPase activity. As a working hypothesis, we propose that this mechanism may be important in physiological events linked to MOM externalization of CL accompanied with increased mitochondrial fission including apoptosis or mitophagy [51], [52]. In conclusion, the current study points to CL as an important regulator of Drp1's activity and consequently of mitochondrial fission.
